# Male age and its association with reproductive traits in captive and wild house sparrows

**DOI:** 10.1111/jeb.13542

**Published:** 2019-09-26

**Authors:** Antje Girndt, Glenn Cockburn, Alfredo Sánchez‐Tójar, Moritz Hertel, Terry Burke, Julia Schroeder

**Affiliations:** ^1^ Research Group Evolutionary Biology Max Planck Institute for Ornithology Seewiesen Germany; ^2^ Department of Life Sciences Imperial College London Silwood Park Campus UK; ^3^ Department of Evolutionary Biology Bielefeld University Bielefeld Germany; ^4^ Research Group Evolution of Sensory Systems Max Planck Institute for Ornithology Seewiesen Germany; ^5^ Department of Behavioural Neurobiology Max Planck Institute for Ornithology Seewiesen Germany; ^6^ Department of Animal and Plant Sciences University of Sheffield Sheffield UK

**Keywords:** extra‐pair paternity, gamete selection, internal fertilization, multiple mating, polygamy, sperm competition

## Abstract

Evolutionary theory predicts that females seek extra‐pair fertilizations from high‐quality males. In socially monogamous bird species, it is often old males that are most successful in extra‐pair fertilizations. Adaptive models of female extra‐pair mate choice suggest that old males may produce offspring of higher genetic quality than young males because they have proven their survivability. However, old males are also more likely to show signs of reproductive senescence, such as reduced sperm quality. To better understand why old males account for a disproportionally large number of extra‐pair offspring and what the consequences of mating with old males are, we compared several sperm traits of both captive and wild house sparrows, *Passer domesticus*. Sperm morphological traits and cloacal protuberance volume (a proxy for sperm load) of old and young males did not differ substantially. However, old males delivered almost three times more sperm to the female's egg than young males. We discuss the possibility of a post‐copulatory advantage for old over young males and the consequences for females mated with old males.

## Graphical abstract

We found that old male house sparrows delivered almost three times more sperm to a female's egg than young males. Our result supports the observation that old males robustly gain more extra‐pair paternity, and we discuss the reproductive consequences for females mated with old males.

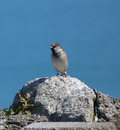

## 1 INTRODUCTION

In socially monogamous mating systems, mating outside the pair bond (i.e. extra‐pair mating) is adaptive for females if females gain direct (e.g. access to resources) or indirect (i.e. genetic) benefits (Griffith, Owens, & Thuman, 2002). In birds, male age is a robust predictor of extra‐pair paternity (Cleasby & Nakagawa, 2012). Models of female choice support a preference for old males because old males have proven their viability, and female preference for old males could evolve if female preference is heritable and male viability is passed on to genetic offspring (Kokko & Lindstrom, 1996; Manning, 1985). Additionally, old males may be ageing or senescent males, which means that their sperm—the only direct benefit passed on in an extra‐pair mating—will be of lower quality (Kong et al., 2012; Pizzari, Dean, Pacey, Moore, & Bonsall, 2008). A premeiotic age‐related reduction in sperm quality could incur direct (e.g. reduced fertilizing efficiency) and indirect (e.g. decreased offspring fitness) costs to females mated to old males (Pizzari et al., 2008). For instance, in insemination experiments in houbara bustards, *Chlamodytis undulata*, advanced paternal age was linked with inhibited post‐hatching offspring growth (Preston, Saint Jalme, Hingrat, Lacroix, & Sorci, 2015). Advanced paternal age was also associated with lower lifetime reproductive fitness in a wild house sparrow, *Passer domesticus*, population (Schroeder, Nakagawa, Rees, Mannarelli, & Burke, 2015). Indeed, females suffering lower fecundity or lower quality offspring is a prediction of the polyandry hypothesis contrasting the above‐described models of female choice for old males (Radwan, 2003). The polyandry hypothesis suggests that females opt for extra‐pair mating to avoid fertilizations by old males. The hypothesis predicts further that females are indifferent to male age during mate choice and old males are worse sperm competitors than young males (Radwan, 2003). A recent study found no evidence that female house sparrows preferred old males for mating (Girndt, Chng, Burke, & Schroeder, 2018) but, like in other birds, old captive and wild house sparrow males also achieve most extra‐pair paternity (Girndt et al., 2018; Hsu, Schroeder, Winney, Burke, & Nakagawa, 2015). These are intriguing findings because if old males achieve most extra‐pair paternity but are not preferred in extra‐pair matings, it is unlikely that old males are worse sperm competitors than young males like the polyandry hypothesis suggests. Instead, old males might have a post‐copulatory advantage over young males.

Sperm quantity (e.g. sperm number) and sperm quality (e.g. morphology) are important for male reproductive success, and scientific knowledge about the effects of male age on sperm traits is rapidly growing. Meta‐analytical evidence showed that sperm quality decreases with increasing male age in humans, *Homo sapiens* (Johnson, Dunleavy, Gemmell, & Nakagawa, 2015), and a similar trend has been found in brown Norway rats, *Rattus norvegicus* (Syntin & Robaire, 2001); blue‐footed boobies, *Sula nebouxii* (Velando, Noguera, Drummond, & Torres, 2011); barn swallows, *Hirundo rustica* (Møller et al., 2009); and red junglefowl, *Gallus gallus* (Dean et al., 2010). However, if sperm quality decreases with age, maybe other post‐copulatory traits are at work for old males to sire a disproportionally large number of extra‐pair offspring. What if old males, while producing lower quality sperm, have increased sperm production? A higher number of sperm could give old males a numerical advantage over young males during sperm competition despite the overall lower quality of their sperm (Parker, 1990).

Increased sperm production by old males has been observed in internally and externally fertilizing fish (e.g. Gasparini, Marino, Boschetto, & Pilastro, 2010; Mehlis & Bakker, 2013; Vega‐Trejo, Fox, Iglesias‐Carrasco, Head, & Jennions, 2019). In humans, male age and sperm number do not seem to be associated (Johnson et al., 2015). In birds, there are hints of sperm number being associated with male age when testes size is considered to be a proxy for sperm quantity (De Reviers & Williams, 1984; Sax & Hoi, 1998). Male birds in their first year of breeding have testes that are approximately 27% smaller than testes of older breeders (Calhim & Birkhead, 2007). Also, male passerines develop a cloacal protuberance indicative of their reproductive status (Wolfson, 1952), relative testes size and capacity to store sperm (Birkhead, Briskie, & Møller, 1993). The larger a male's cloacal protuberance, the larger his relative testes size and hence sperm reservoir (Birkhead et al., 1993). Again, older males have a larger cloacal protuberance. In two Australian fairywren species, *Malurus lamberti* and *splendens*, older males had larger cloacal protuberances than first‐year breeders, and sperm number correlated positively with cloacal protuberance size (Tuttle, Pruett‐Jones, & Webster, 1996; but see Quay 1986). Cloacal protuberances were also larger in older reed buntings, *Emberiza schoeniclus*, and increased in size with age within males (Bouwman, van Dijk, Wijmenga, & Komdeur, 2007). Collectively, these findings provide support for age‐related variation in reproductive traits and are consistent with the observation that old males robustly gain more extra‐pair paternity across bird species (Cleasby & Nakagawa, 2012).

In house sparrows, it is unclear what sperm phenotype maximizes fertilizing capacity. One study concluded that sperm with relatively short heads swam fastest, and sperm length was positively associated with sperm longevity (Helfenstein, Podevin, & Richner, 2010), but no such association was found in another study (Cramer et al., 2015). Sexual selection will favour sperm phenotypes that can both outcompete rival's sperm (e.g. be the fastest sperm [Knief et al., 2017]) and avoid being outcompeted (Birkhead, 1989; e.g. avoid oxidative stress [Mora, Firth, Blareau, Vallat, & Helfenstein, 2017]). Therefore, multiple sperm traits will affect sperm performance and multiple sperm traits need to be analysed to understand differences in sperm competitiveness.

Here, we tested the hypothesis that post‐copulatory competitiveness changes with age in captive and wild house sparrows. Our specific aims were to test: (a) whether sperm length is associated with male age, without predicting directionality; and (b) whether the proportion of morphologically abnormal sperm is higher in old compared to young males. Further, to indirectly assess whether old males provide more sperm than young males, we studied (c) cloacal protuberance volume and (d) the number of sperm trapped on egg membranes (i.e. perivitelline layers, hereafter PVL; Wishart, 1987). In birds, the egg is surrounded by the PVL and the number of sperm at the PVL exemplifies the number of inseminated sperm and the probability of an egg being fertilized (Brillard & Antoine, 1990; Froman, Pizzari, Feltmann, Castillo‐Juarez, & Birkhead, 2002; Wishart, 1987). Although PVL sperm are a useful noninvasive proxy for the number of inseminated sperm and monitoring fertility in a pair (Croyle, Durrant, & Jensen, 2015), the dynamics behind the dramatic reduction in sperm number from the cloaca to the egg (Bakst, Wishart, & Brillard, 1994) are complex and not well understood (Birkhead & Brillard, 2007). Various reasons such as interactions between sperm phenotype and the female sperm storage tubules or vaginal sperm selection (Hemmings, Bennison, & Birkhead, 2016) add to explain variation in the number of sperm that reach the egg.

## 2 MATERIALS AND METHODS

### 2.1 Captive house sparrows

House sparrows were kept at the Max Planck Institute for Ornithology in Seewiesen, Germany (47.9752°N, 11.2332°E), since 2005. The cohorts of 2005 and 2006 were wild‐caught birds from rural Bavaria (Laucht, Kempenaers, & Dale, 2010), and breeding took place in most of the subsequent years. All birds were fitted with a unique numbered metal ring and combination of colour rings for identification. The specific husbandry under semi‐natural conditions has been described and illustrated previously (Girndt et al., 2017, 2018).

### 2.2 Wild house sparrows

The wild house sparrows are resident on Lundy Island, approximately 19 km off the coast of Devon, England (51.1781°N, 4.6673°W). The population has been systematically monitored since 2000 allowing for individual identification and knowledge of precise individual ages, and social and genetic pedigrees. Annual resighting rates are 91%–96%, and migration to and from the mainland is almost absent (Schroeder, Cleasby, Nakagawa, Ockendon, & Burke, 2011; Simons, Winney, Nakagawa, Burke, & Schroeder, 2015).

### 2.3 Sperm collection techniques

Sperm were collected during the reproductive season of house sparrows (March until August; Anderson, 2006) in 2014 and 2015. Sperm were obtained using the standard techniques of faecal and abdominal massage sampling, which we have described and illustrated in depth previously (Girndt et al., 2017). Briefly, samples were stored in 200 μl of 5% formalin before placing 10 μl aliquots onto microscope slides for morphological assessment of sperm. House sparrow males replenish their ejaculates overnight (Birkhead, Veiga, & Møller, 1994b). In captivity, we isolated males and females for at least 2 days before sperm collection to standardize samples for males’ mating histories, which affect post‐meiotic sperm senescence independent of male age (Pizzari et al., 2008; Vega‐Trejo et al., 2019). In the wild, males could not be isolated from females, and we only applied abdominal massage to collect sperm.

### 2.4 Length of sperm components

Sperm linear measurements were as described (Girndt et al., 2017). Briefly, we took digital images of the first ten intact (i.e. no broken tails or heads), unobstructed (i.e. not covered by detritus) and morphologically normal sperm (see the abnormality section below for a definition). We always started in the upper left corner of the microscope slide using a Leica DFC450‐C camera mounted on a Zeiss Axioplan 2 microscope at 400× magnification (40× objective) in bright field settings. Sperm components (i.e. head including acrosome, flagellum including midpiece) were measured from digital images using the Leica Application Suite software v4.2. by one observer only (GC), who was blind regarding sample identities. Total length was calculated as the sum of the head and flagellum measures, and mean observer repeatability was high for all sperm components (*R* > 0.82; Girndt et al., 2017).

### 2.5 Proportion of morphologically abnormal sperm

Sperm were classified as abnormal if they deviated from the typical passerine (oscine) shape, which consists of an acrosome, a nucleus and a flagellum, consisting of the midpiece whose mitochondria form a helix around the axoneme and the nonhelical tail (Aire, 2007). Abnormalities affected all sperm components, such as sperm heads (e.g. bends of more than 90°), midpieces (e.g. distal cytoplasmic droplets) and tails (e.g. coiled, stubbed or super numerous). Sperm abnormality screening of the first 100 intact and unobstructed sperm was done by one observer only (AG), always starting in the upper left corner of each microscope slide. To establish observer repeatability, a subset of 20 microscope slides was randomly selected using the function sample in R version 3.5.3 (R Development Core Team, 2013). Sperm were then screened again, following the same protocol, so that the individual sperm measured were identical on both occasions. However, the microscopes used differed between the two occasions. Although we mostly used the Zeiss Axioplan 2 microscope, we also relied on a substitute, Olympus BX50, microscope. Observer repeatability (here and all following data) was calculated using the R package rptR v. 0.9.2 (Stoffel, Nakagawa, & Schielzeth, 2017) in R version 3.5.3 (R Development Core Team, 2013). Because the second microscope introduced variation to the data, we added it as a fixed effect to calculate adjusted observer repeatability for abnormality scores. Adjusted observer repeatability was high: *R* = 0.78 ± 0.11 standard error (*SE*) (95% CI (confidence interval): 0.50–0.94, *p *<* *.0001) (see the Supplements for the unadjusted observer repeatability analysis). Further, the observer could guess the age of some captive but never wild males from the sample descriptions but attempted to hide descriptions from view when scoring abnormal sperm to be blind in the majority of the measurements.

### 2.6 Cloacal protuberance volume

The diameter and height of the cloacal protuberance were measured with callipers to the nearest 0.1 mm by one observer per population. Measurements took place before abdominal massages were applied (Quay, 1986). We used the cone formula ($\frac{1}{3}\pi r^{2}h$, *r = c*loacal protuberance width/2, *h = c*loacal protuberance height) to calculate cloacal protuberance volume because a cone best describes the shape of the cloacal protuberance of house sparrows (Wolfson, 1952). The observer remeasured 136 captive males, kept in single‐sex aviaries within 48 hr, expecting cloacal protuberance size to be stable during that period (i.e. we expected absent or negligible within‐individual variance in cloacal protuberance during that period), and estimated observer repeatability, which was high: *R* = 0.73 ± 0.04 *SE* (95% CI: 0.64 to 0.80, *p *<* *.001). Observer repeatability for the wild house sparrows could not be estimated because of insufficient repeat measurements (e.g. six recaptures in 2015 with the shortest being 28 days apart). Both observers measured the same 12 captive house sparrows once each to estimate repeatability, which was also high: (*R* = 0.76 ± 0.14 *SE* (95% CI: 0.38 to 0.92), *p *=* *.004).

### 2.7 Sperm on PVL

We collected unincubated eggs from captive females that were held in aviaries with only either old males (7 and 8 years old) or young males (1 and 3 years old). We did not collect eggs from the wild population. Our aviary set‐up (*N *=* *9 aviaries) ensured that eggs could only have been fertilized by males of one age group, dependent on the aviary in which the egg was laid. Note that 3‐year‐old house sparrows would be considered ‘mature’ in the wild (e.g. less than 20% of wild house sparrows survive until 3 years of age) but can be considered young in captivity where mortality is comparably lower (Simons et al., 2019). Lower mortality in captivity leads to birds growing older and the absence of a typical age‐structured pyramid with more first‐year than older breeders. For instance, 57% of the captive males used for sperm linear analysis were older than 3 years (see data at the open science framework). Aviaries held eight to nine pairs of birds, apart from one aviary with 13 pairs. We counted sperm on the PVL and examined the fertilization status of 41 nonincubated eggs following an established protocol (Birkhead, Hall, Schut, & Hemmings, 2008). We did not count holes made by sperm hydrolysing the PVL because the number of sperm on the PVL correlates with the number of holes (Birkhead, Sheldon, & Fletcher, 1994a). We carefully opened eggs with scissors, removed the germinal disc and washed it with phosphate‐buffered saline (PBS). We put the germinal disc on a microscope slide, added a drop of DNA stain Hoechst 33342 (0.05 mg/ml) and searched for diploid cells as evidence of fertilization (Birkhead et al., 2008) with the Zeiss Axioplan 2 microscope in fluorescent mode. Next, we removed the PVL from the yolk, washed it in PBS and stretched the entire PVL onto a microscope slide. We again added a few drops of Hoechst and systematically counted fluorescent sperm nuclei using the same microscope and a tally counter. Eggs were prepared and examined by one observer only (AG), who was blind towards the experimental age treatment.

### 2.8 Statistical analyses

We ran statistical models using R version 3.5.3 (R Development Core Team, 2013) and the package lme4 version 1.1‐21 (Bates, Mächler, Bolker, & Walker, 2014). We used the package arm version 1.10‐1 and the function sim (Gelman & Hill, 2007) to simulate values from the posterior distributions (*N *=* *2,000 draws) of the model parameters. Throughout, we used noninformative priors. From the simulated values, we extracted 95% credible intervals (CrI). CrI not overlapping zero can be interpreted as a frequentist *p *<* *.05 (Korner‐Nievergelt et al., 2015). In line with recent calls to improve statistical inference, we decided to report our observed effects as continuous measures of strength of evidence against the null hypothesis (Amrhein, Greenland, & McShane, 2019; Amrhein, Korner‐Nievergelt, & Roth, 2017), using the language of the ‘statistical clarity concept’ (Dushoff, Kain, & Bolker, 2019), instead of emphasizing statistically significant results.

For all models, we followed recommendations to ensure that model assumptions were met, including ruling out overdispersion in non‐Gaussian models and multi‐collinearity between predictors (Korner‐Nievergelt et al., 2015). In all models, continuous variables (e.g. male age, day of year) were mean‐centred and scaled, so that the variables were measured in the unit of standard deviations (*SD*) from the mean. We specifically refer to either the captive or the wild house sparrow data set when describing our statistical model structure, unless the model structure was identical for both populations.

#### 2.8.1 Length of sperm components

We fitted linear mixed models with the total length of single sperm components as the response variable. We used individual data from all sperm measured per male (range 10–30 sperm per male) instead of using means or medians of sperm length. Male age in years was an explanatory variable. Further, we estimated standardized multi‐locus heterozygosity (hereafter sMLH) as a proxy for the degree of inbreeding from genetic marker data, using the R package inbreedR version 0.3.2 (Stoffel et al., 2016), to account for potential inbreeding affecting sperm morphology. The identity and details of the genetic markers were published previously (Dawson et al., 2012; Girndt et al., 2018). We added sampling years (levels: 2014 and 2015) and the method of sperm collection (captive house sparrow data only) as explanatory variables (levels: abdominal massage and faeces). Further, captive male house sparrows were either assigned or not to mixed‐sex aviaries (*N *=* *16 aviaries), which created a sperm competition environment only for those males in mixed‐sex aviaries because males in male‐only aviaries could not compete for the fertilization of eggs. We therefore added aviary set‐up (levels: with and without females) as an explanatory variable to the captive data set. We included sample, male and aviary identities as random effects on the intercept to account for the nonindependence of sperm from the same sample, repeated measurements of males and potential aviary grouping effects in the captive house sparrow data set. We measured 3,262 sperm from 127 captive male house sparrows, which were between 1 and 10 years old. For the wild house sparrows, we had 672 sperm available from 34 males aged 1–4 years.

#### 2.8.2 Proportion of morphologically abnormal sperm

Abnormality counts were fitted as a proportional two‐column matrix response variable using cbind in R (i.e. number of abnormal sperm and number of normal sperm) in generalized linear mixed models assuming a binomial error structure. Male age was modelled as an explanatory variable, as well as sMLH. We further fitted the following explanatory variables to the captive data set: aviary set‐up (*N *=* *7 aviaries) (levels: with and without females), sperm collection method (levels: abdominal massage and faeces), and microscope used (levels: Zeiss and Olympus). Male identity was fitted as random effect on the intercept for the analysis of the captivity data to account for repeated measures. Year (levels: 2014 and 2015) was added as an explanatory variable to the wild house sparrow data. Models for both populations were overdispersed (Korner‐Nievergelt et al., 2015), so we added an observation‐level random effect. We had 87 samples available from 73 captive (between 1 and 10 years old) and 23 samples from 23 wild house sparrows (between 1 and 5 years old).

#### 2.8.3 Cloacal protuberance volume

To test for an association of the cloacal protuberance size with age, we fitted cloacal protuberance volume as a response variable in a linear mixed model. We accounted for potential seasonal and body size effects by adding day of the year (captivity: 14–21 June; wild: 6 May–17 August) and tarsus length as continuous explanatory variables. Additionally, a squared day of the year term was fitted for the wild house sparrow data because sampling took place during the whole breeding season, which could have led to nonlinear seasonal changes in cloacal protuberance volume (Anderson, 2006). Further, we included the explanatory variable aviary set‐up (*N *=* *7 aviaries) (levels: with and without females) to the captive house sparrow analysis and year (levels: 2015 and 2016) to the wild house sparrow analysis. Male identity was fitted as random effect on the intercept, but the variance component was estimated as zero for the wild house sparrows. This may mean that we could not fully account for repeated measurements of males. To ensure that the model was robust, we reran it using only one randomly selected observation per male (function sample in R [R Development Core Team, 2013]; Table S3). We had 195 observations from 142 captive (between 1 and 10 years old) and 56 observations from 46 wild house sparrows (between 1 and 5 years old).

#### 2.8.4 Number of sperm on PVL

**Figure 1:**
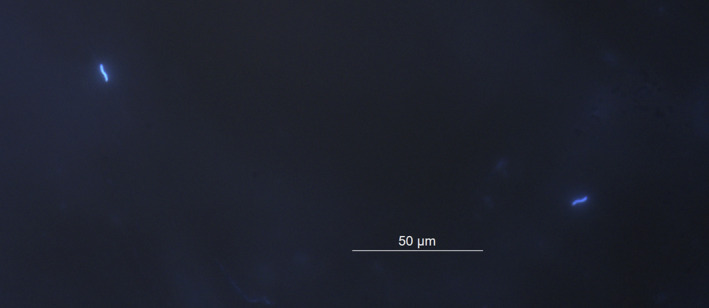
Sperm on the perivitelline layer (PVL). Two fluorescent house sparrow nuclei bound on the perivitelline membrane stained with Hoechst 33342

We show descriptive statistics for the number of sperm on the PVL (Figure 1b). We also ran an unequal variances *t* test to compare the mean number of sperm (log‐transformed) from old and young males at 40 eggs. However, this approach should be treated cautiously because the male sperm donor and, therefore, the possibility of nonindependence of data could not be established. Additionally, sperm counts (*N *=* *40 eggs) were fitted as a response variable in a generalized linear mixed model assuming a Poisson error structure. Male age and female age (levels: old and young) were modelled as explanatory variables and we estimated the percentage of variance explained by male and female age (R^2^_marginal_) following (Nakagawa and Schielzeth, 2013). Aviary (*N *=* *9) was fitted as random effect on the intercept. The model was overdispersed, so we added an observation‐level random effect.

### 2.9 Data statement and accessibility

All data and the R scripts are publicly available at the Open Science Framework (https://doi.org/10.17605/osf.io/pkwsr). We confirm that we have reported all measures, conditions and data exclusions for the questions addressed in this publication. Sample sizes were determined by subject availability.

## 3 RESULTS

### 3.1 Length of sperm components

We did not find a statistically clear effect of male age on the length of sperm components. This was also the case for sMLH (Tables 1 and 2). As previously shown in the captive population (Girndt et al., 2017), sperm sampled from faeces were shorter than sperm sampled by abdominal massage (1). When the analysis was restricted to abdominal massage sampled sperm (2,148 examined sperm from 116 males), the results were qualitatively similar to the main data set analyses, showing no statistical clear relationship between length of sperm components and male age (Table S1). Unexpectedly, and not among this study's original predictions, we further found that sperm were longer in males from mixed‐ than single‐sex aviaries (Table 1). Additionally, we observed statistical effects on sperm length components between years in both populations (Tables 1 and 2).

**Table 1:** Results from a linear mixed model estimating the effect of male age on (a) the total, (b) the head, (c) the midpiece and (d) the flagellum length of 3,262 sperm from 127 captive male house sparrows

Sperm length (μm)	
Captive house sparrows	Estimate (lower CrI to upper CrI)
(a) Total length	
(intercept)	99.48 (98.76 to 100.18)
Age	0.36 (−0.10 to 0.86)
sMLH	−0.09 (−0.55 to 0.34)
Aviary set‐up (with females)	1.06 (0.42 to 1.66)
Method (faeces)	−0.51 (−0.92 to −0.09)
Year (2015)	−0.32 (−0.89 to 0.25)
Random effects	
Male ID	7.15 (5.72 to 8.80)
Aviary	0.04 (0.02 to 0.08)
Sample ID	0.83 (0.70 to 1)
Residual variance	2.88 (2.81 to 2.95)
(b) Head	
(intercept)	14.12 (13.82 to 14.43)
Age	0.06 (−0.08 to 0.19)
sMLH	−0.08 (−0.18 to 0.03)
Aviary set‐up (with females)	0.15 (−0.15 to 0.42)
Method (faeces)	−0.32 (−0.47 to −0.18)
Year (2015)	−0.53 (−0.80 to −0.24)
Random effects	
Male ID	0.25 (0.19 to 0.31)
Aviary	0.03 (0.01 to 0.06)
Sample ID	0.17 (0.15 to 0.219)
Residual variance	0.86 (0.84 to 0.88)
(c) Midpiece	
(intercept)	66.43 (65.86 to 66.99)
Age	0.06 (−0.31 to 0.43)
sMLH	0.12 (−0.21 to 0.45)
Aviary set‐up (with females)	1.01 (0.53 to 1.51)
Method (faeces)	−0.34 (−0.72 to 0.03)
Year (2015)	0.98 (0.51 to 1.46)
Random effects	
Male ID	4.19 (3.37 to 5.08)
Aviary	0.02 (0.01 to 0.03)
Sample ID	0.64 (0.53 to 0.76)
Residual variance	2.71 (2.65 to 2.77)
(d) Flagellum	
(intercept)	85.45 (84.72 to 86.15)
Age	0.24 (−0.21 to 0.70)
sMLH	0 (−0.44 to 0.41)
Aviary set‐up (with females)	0.86 (0.28 to 1.46)
Method (faeces)	−0.19 (−0.55 to 0.18)
Year (2015)	0.14 (−0.43 to 0.70)
Random effects	
Male ID	7.40 (5.93 to 9.02)
Aviary	0.07 (0.03 to 0.14)
Sample ID	0.51 (0.42 to 0.60)
Residual variance	2.80 (2.73 to 2.86)

Note

We accounted for standardized multi‐locus heterozygosity (sMLH), aviary set‐up (levels: with and without females), sperm collection method (levels: abdominal massage and faeces) and year (levels: 2014 and 2015) of sperm collection. Male age, as well as sMLH, was centred and scaled. We present posterior means and CrI (95% credible interval).

**Table 2:** Results from a linear mixed model estimating the effect of male age on (a) the total, (b) the head, (c) the midpiece and (d) the flagellum length from 672 sperm of 34 wild male house sparrows

Sperm length (μm)	
Wild house sparrows	Estimate (lower CrI to upper CrI)
(a) Total length	
(intercept)	99.22 (98.06 to 100.35)
Age	−0.07 (−1.03 to 0.90)
sMLH	0.52 (−0.51 to 1.58)
Year (2015)	−2.81 (−4.44 to −1.22)
Random effects	
Male ID	9.14 (7.26 to 11.80)
Residual variance	2.60 (2.47 to 2.74)
(b) Head	
(intercept)	13.10 (12.82 to 13.39)
Age	−0.05 (−0.30 to 0.19)
sMLH	0.13 (−0.11 to 0.37)
Year (2015)	−0.29 (−0.73 to 0.17)
Random effects	
Male ID	0.57 (0.47 to 0.70)
Residual variance	0.82 (0.78 to 0.86)
(c) Midpiece	
(intercept)	68.02 (67.35 to 68.66)
Age	0.40 (−1.08 to 0.07)
sMLH	−0.52 (−0.31 to 0.42)
Year (2015)	−0.10 (−1.22 to 1.10)
Random effects	
Male ID	2.64 (2.03 to 3.35)
Residual variance	2.66 (2.52 to 2.81)
(d) Flagellum	
(intercept)	86.06 (85.01 to 87.17)
Age	0.05 (−0.83 to 0.91)
sMLH	0.38 (−0.52 to 1.26)
Year (2015)	−2.33 (−3.82 to −0.87)
Random effects	
Male ID	7.30 (5.71 to 9.28)
Residual variance	2.57 (2.44 to 2.71)

Note

We accounted for sMLH and year of sperm collection (levels: 2014 and 2015). Male age, as well as sMLH, was centred and scaled. We present posterior means and CrI.

### 3.2 Proportion of morphologically abnormal sperm

Captive house sparrows had on average 16.8% ± 12.9 (mean ± *SD*,* N *=* *87 samples) morphologically abnormal sperm, compared to 5.3% ± 8.7 (*N *=* *23 samples) morphologically abnormal sperm in the wild house sparrows, which was a substantial difference (χ^2^ = 5.68, *df* = 1, *p *=* *.02). In neither data set did the proportion of morphologically abnormal sperm and male age show a clear statistical relationship (3). The statistical model on the wild house sparrow data was overfitted, which can lead to type 1 errors (Forstmeier, Wagenmakers, & Parker, 2016). Because we interpreted our result as a lack of statistical association between the proportion of abnormal sperm and male age (Table 3b), we can rule out that the result is a type 1 error.

**Table 3:** Results from a generalized linear mixed model on the proportion of morphologically abnormal sperm in relation to male age in captive (87 samples of 73 males) and wild house sparrows (23 samples of 23 males)

Proportion of morphologically abnormal sperm (logit‐link scale)	
	Estimate (lower CrI to upper CrI)
(a) Captive house sparrows	
(intercept)	−2.24 (−2.66 to −1.84)
Age	0.16 (−0.06 to 0.38)
sMLH	−0.09 (−0.33 to 0.12)
Aviary set‐up (with females)	0.15 (−0.58 to 0.80)
Method (faeces)	−0.09 (−0.56 to 0.37)
Microscope (Olympus)	0.77 (0.11 to 1.44)
Random effects	
Male ID	0.26 (0.18 to 0.36)
Aviary	0 (0 to 0)
Observation‐level random effect	0.57 (0.43 to 0.73)
(b) Wild house sparrows	
(intercept)	−3.84 (−4.50 to −3.16)
Age	0.22 (−0.39 to 0.83)
sMLH	0.62 (−0.07 to 1.31)
Year (2015)	0.44 (−1.01 to 1.90)
Random effects	
Observation‐level random effect	1.73 (1.14 to 2.49)

Note

We accounted for sMLH in both populations, aviary set‐up (levels: with and without females), sperm collection method (levels: abdominal massage and faeces), the microscope used (levels: Zeiss and Olympus) in the captive house sparrows and year (levels: 2014 and 2015) in the wild house sparrows. Male age, as well as sMLH, was centred and scaled. We present posterior means and CrI.

The Olympus microscope caused a statistical upward bias of abnormality scores in the captive population (Table 3). When we restricted the data set to the main, Zeiss, microscope (51 samples of 38 males instead of 87 samples of 73 males), our interpretation of no clear statistical relationship between the proportion of morphologically abnormal sperm and male age remained qualitatively similar (Table S2).

### 3.3 Cloacal protuberance volume

There was no apparent statistical association between cloacal protuberance volume and male age in either population. This was also the case for sMLH (both populations), the aviary set‐up (captive population), method of sampling (captive population) and the year sampling took place (wild population). We further found a large among‐male variance in the captive population (Table 4). Cloacal protuberance volume showed a positive statistical association with tarsus size and day of the year in captivity (Table 4). In the wild, cloacal protuberance volume showed a negative statistical association with the day of sampling, highlighting a seasonal decrease (Table 4).

**Table 4:** Results from a linear mixed model on cloacal protuberance volume (mm^3^) in relation to male age in captive (195 observations of 142 males) and wild house sparrows (56 observations of 46 males)

Cloacal protuberance volume (mm^3^)	
	Estimate (lower CrI to upper CrI)
(a) Captive house sparrows	
(intercept)	49.37 (42.05 to 57.03)
Age	−1.07 (−4.43 to 2.34)
Aviary set‐up (with females)	2.57 (−7.91 to 13.90)
Day of year	4.13 (0.60 to 7.49)
Tarsus	2.86 (0.06 to 5.64)
Random effects	
Male ID	222.69 (184.93 to 264.59)
Aviary	15.12 (4.45 to 31.69)
Residual variance	9.03 (8.19 to 9.97)
(b) Wild house sparrows	
(intercept)	3.41 (3.12 to 3.68)
Age	0.10 (−0.07 to 0.26)
Day of year	−0.17 (−0.51 to 0.15)
Day of year^2^	−0.20 (−0.46 to 0.06)
Tarsus	−0.04 (−0.21 to 0.12)
Year (2016)	−0.04 (−0.54 to 0.47)
Random effects	
Male ID	0 (0 to 0)
Residual variance	0.61 (0.50 to 0.75)

Note

We accounted for day of the year (captivity: 14–21 June; wild: 6 May–17 August) and tarsus size in both populations. Aviary set‐up (levels: with and without females) was added to the analysis on captive house sparrows, and year (levels: 2015 and 2016) was added to the analysis on wild house sparrows. Cloacal protuberance volume of wild house sparrows was log‐transformed.

### 3.4 Number of sperm on PVL

**Figure 2:**
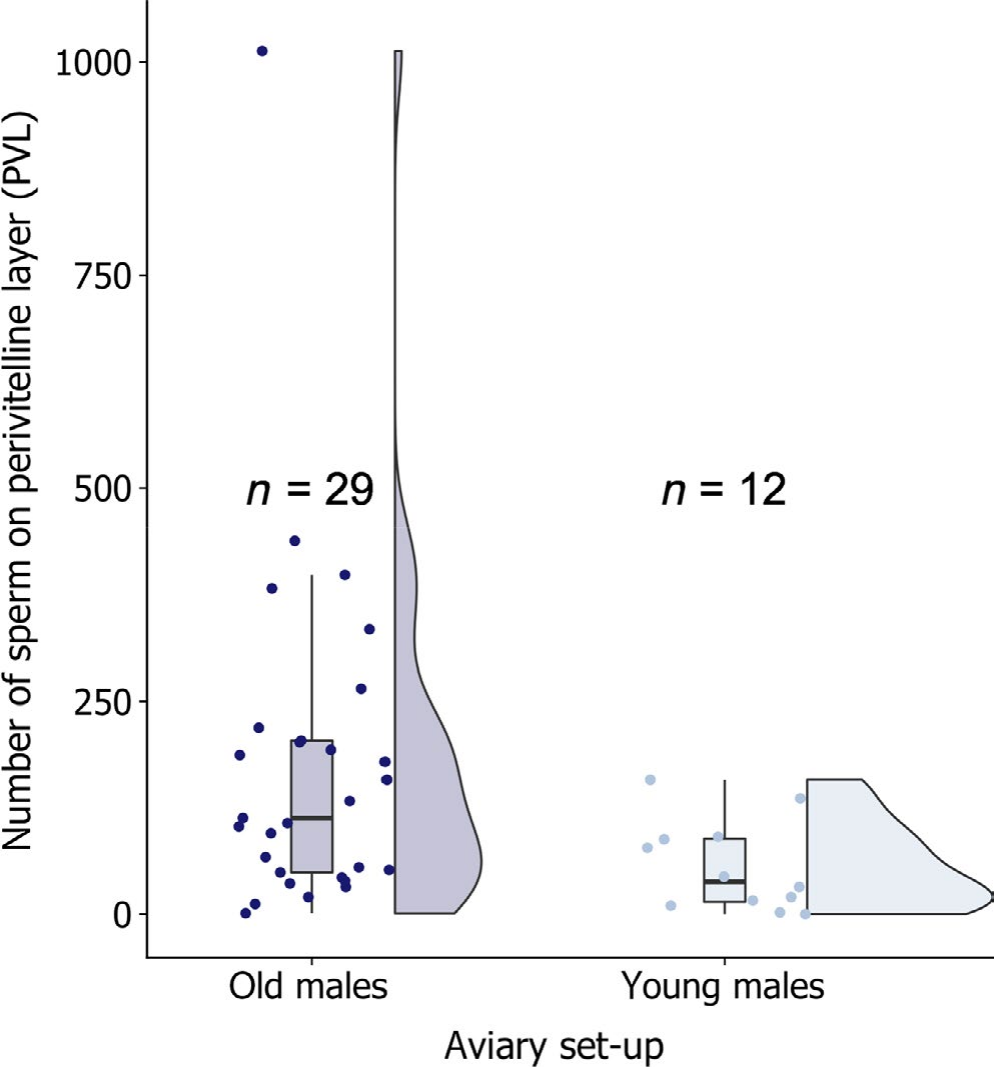
The effect of age treatment on the number of sperm on the PVL. The number of sperm on perivitelline layers (PVL) of 41 eggs was approximately three times higher in aviaries with old (>6 years) than aviaries with young males (1–3 years). We visualized the raw data including an outlier (one egg with 1,013 sperm) using a raincloud plot, combining box, split violin and scatter plots (Allen, Poggiali, Whitaker, Marshall, & Kievit, 2019). The outlier was not included in statistical analyses

The number of sperm counted ranged from 0 to 1,013 (1 for an example of two sperm on a PVL). The mean number of old males’ sperm reaching the eggs of females (mean ± *SD*: 147 ± 124, *N *=* *28 eggs) was nearly three times higher than the mean number of young males’ sperm (56 ± 53, *N *=* *12 eggs, Figure 2), which was a considerable difference (unequal variances *t* test, *t*_16.73_ = 2.36, *p *=* *.03, supplementary analysis [Table S4]). Male age explained 16.4% of the variance and female age 0 (R^2^ _marginal_). We excluded an outlier egg with 1,013 sperm (*z*‐score = 7, so 7 *SD* above the mean value of all sperm counted) from the *t* test (Figure 2). Including it would have strengthened the result. Further, of 41 eggs examined, 39 were fertilized. The two unfertilized eggs originated from an aviary of each male age group.

## 4 DISCUSSION

Our overall aim was to elucidate the factors promoting a positive relationship between extra‐pair paternity and male age. Specifically, we predicted a sperm quantity–quality trade‐off related to male age. However, we found no evidence for such a trade‐off in two populations of house sparrows. Specifically, we did not find a clear statistical association of sperm morphology or cloacal protuberance size with male age. Instead, we found that in captivity, the number of old males’ sperm in the eggs of females was almost three times higher than the number of young males’ sperm. Our result is intriguing because neither the number of mating attempts, the number of copulations nor female choice are explained by male age in this population (Girndt et al., 2018). Hence, precopulatory differences do not seem to explain the age‐related difference in extra‐pair copulation success and it is tempting to suggest age‐related post‐copulatory differences between old and young males. Old males might have inseminated more sperm, and/or there was cryptic female choice (Eberhard, 2009) of sperm from old males. Yet, our result is limited by a lack of information on the identities of the males that provided the sperm. For example, did all males in each aviary inseminate females? Also, whether more sperm on PVLs constitute a curse or a blessing remains to be seen too. This is because the more the sperm are inseminated, the higher the probability that the egg gets fertilized (Brillard & Antoine, 1990; Froman et al., 2002; Wishart, 1987), but the risk of embryo mortality caused by multiple sperm entering the egg (i.e. polyspermy; Forstmeier & Ellegren, 2010) might also be elevated. In our study, 95% of eggs were fertilized (*N *=* *41 eggs total) pointing at two things. First, there was no difference in the fertilizing ability of young and old males. Second, infertility was rare (Schmoll & Kleven, 2016). Indeed, in house sparrows, the biggest cause of unhatched eggs is embryo mortality (Birkhead, Veiga, & Fletcher, 1995). Under the assumption that old males inseminate more sperm, this could mean that they outcompete young males with sperm numbers in sperm competition (Parker, 1990), at the cost of an elevated risk of unhatched eggs. Subsequent efforts could investigate the idea of such a double‐sided effect of male age.

Cloacal protuberance volume was positively associated with tarsus size, as well as date of measurement in captive house sparrows, whereas it was negatively associated with the date of measurement in the wild house sparrows. In the wild, measurements included the end of the breeding season, so the decline in cloacal protuberance volume can be interpreted as the regression of male reproductive gonadal growth (Anderson, 2006; Sax & Hoi, 1998). We also found a large among‐male variance in cloacal protuberance volume in the captive males, emphasizing that individual‐level predictors other than age and body size must be at play. It would be worthwhile to analyse other individual‐level predictors, such as individual mating status, in the future (Sax & Hoi, 1998).

There is evidence from nonavian studies for a positive association between sperm length and male age (Gasparini et al., 2010; Green, 2003), but the lack of a clear statistical association between sperm length and male age in our data corroborates the results in other passerines with less precise age information (Cramer, Laskemoen, Kleven, & Lifjeld, 2013; Laskemoen, Fossøy, Rudolfsen, & Lifjeld, 2008; Møller et al., 2009). Our results further revealed differences in sperm length in relation to the year of sampling (a), the social environment (b) and the method of sperm sampling (c). (a) The result of differences in sperm length across years might reflect an underlying seasonality. House wrens, *Troglodytes aedon* (Cramer et al., 2013), and male red‐winged blackbirds, *Agelaius phoeniceus* (Lüpold, Birkhead, & Westneat, 2012), show seasonal changes in sperm length. In the latter population, sperm length additionally varied across years (Lüpold et al., 2012). (b) We found that males kept with females had longer midpieces and flagella than males kept with males only. This could indicate a plastic male response to sperm competition, similar to that observed in Gouldian finches, *Erythrura gouldiae,* that increased their midpiece size in high‐competition environments (Immler, Pryke, Birkhead, & Griffith, 2010). Indeed, the social environment affects reproductive development in house sparrows, with males exhibiting declining sperm production and testes degeneration when caged individually (Lombardo & Thorpe, 2009). Also, house sparrows’ midpiece size shows only weak repeatability (Helfenstein et al., 2010), which might support the idea of a plastic response to the social environment. What is unclear is how longer midpieces and flagella affect a sperm's fertilization success because, whereas sperm with longer midpieces and flagella make the best swimmers with the highest fertilization success in zebra finches, *Taeniopygia guttata* (Knief et al., 2017)^,^ in house sparrows, midpiece length and sperm velocity seem to be negatively correlated (Cramer et al., 2015). (c) Additionally, sperm length varied within males in relation to sperm collection method, which is discussed in detail elsewhere (Girndt et al., 2017).

The proportion of morphologically abnormal sperm did not show a statistically clear association with male age. This was surprising because we had relatively many old house sparrows (47 captive males older than 5 years) available and these males are expected to have more mutations in their germline than young males (Kong et al., 2012). Yet, our sample size is modest compared to a study using a breeding facility of 1,080 houbara bustards, where, in males beyond their prime, male age and the proportion of abnormal sperm were positively associated (Preston, Jalme, Hingrat, Lacroix, & Sorci, 2011). Although sperm morphology is an important factor to evaluate a male's fertilization efficiency (Preston et al., 2015), it is also a highly complex trait that is difficult to standardize (Sikka & Hellstrom, 2016). One reason is its sensitivity to an apparatus as simple as a microscope, as evidenced in our results. It is thus possible that other analytical approaches, such as sperm DNA integrity or oxidative stress status assays (Sikka & Hellstrom, 2016), are better suited to detect qualitative differences in sperm of old and young males.

To conclude, sperm morphologies important for fertilization success were unrelated to male age in captive and wild house sparrow. Morphologically abnormal sperm, exemplifying lower quality sperm (du Plessis & Soley, 2011), did not show a clear statistical relationship to male age either, and male's cloacal protuberance sizes were suggestive of similar relative testes sizes and sperm reservoirs in old and young house sparrows. Importantly, the number of sperm reaching the site of fertilization suggested that PVL sperm number and male age were positively correlated, but more sperm at the PVL did not translate into a higher number of eggs being fertilized. Age‐related variation in sperm traits could play an important role in the evolution of polyandry. Contrary to models of female choice for old age, it has been suggested that female extra‐pair mating evolved to help females avoid fertilizations by senescent males (Radwan, 2003). This idea is plausible under the scenario that old males are worse sperm competitors than younger males (Radwan, 2003). Our data do not seem to support this prediction because post‐copulatory traits were mostly similar between old and young male house sparrows and old males might even outcompete young males by sperm number at the site of fertilization. Our study is therefore not only an important step towards elucidating post‐copulatory traits of old versus young male passerines but also towards a better understanding of female polyandry in mating systems where extra‐pair males provide no other direct benefits than sperm. Future data will reveal if conditions are met for adaptive interpretations of female extra‐pair mating with old males or if mating with old males bears a cost.

## CONFLICT OF INTEREST

The authors declare no conflict of interest regarding the publication of this article.

## AUTHOR CONTRIBUTIONS

AG and JS conceived the study. AG and AST carried out sample collection and cloacal protuberance measurements; GC measured all sperm; MH supported the laboratory work and TB the molecular work; and AG scored sperm abnormalities, performed fertilization assays and statistical analysis with support from AST and wrote the manuscript with the help of all co‐authors.
