# Supplementary material for: Male age and its association with reproductive traits in captive and wild house sparrows
Source: J Evol Biol. 2019 Sep 26;32(12):1432–43. doi: 10.1111/jeb.13542 (PMC8653889; doi:10.1111/jeb.13542)
Supplement: Supplementary file 1 [file JEB-32-1432-s001.docx]

**Supplements**

**Observer repeatability abnormality scores**

Observer repeatability was calculated using the R package rptR v. 0.9.2 (Stoffel et al., 2017) in R version 3.5.3 (‘R Development Core Team’, 2013). When not adjusting for the second microscope (see main text) observer repeatability was moderate: R = 0.52 ± 0.16 standard error (SE) (95% CI (Confidence Interval): 0.17 to 0.79, *P* = 0.003).

**Table S1.** Results from a linear mixed model estimating the effect of male age on a) the total, b) the head, c) the midpiece and d) the flagellum length from 2148 sperm of 116 captive male house sparrows using only samples collected via abdominal massage.

| **Sperm length (μm)** | |
| --- | --- |
| **Captive house sparrows** | estimate (lower CrI to upper CrI) |
| **a) total length** |  |
| (intercept) | 99.73 (99 to 100.45) |
| age | 0.24 (-0.22 to 0.73) |
| sMLH | -0.19 (-0.63 to 0.25) |
| aviary set-up (with females) | 0.81 (0.22 to 1.40) |
| year (2015) | -0.53 (-1.09 to 0.03) |
| **Random effects** |  |
| male ID | 6.92 (5.46 to 8.52) |
| aviary | 0 (0 to 0) |
| sample ID | 0.71 (0.56 to 0.89) |
| residual variance | 2.97 (2.86 to 3.06) |
|  |  |
| **b) head** |  |
| (intercept) | 14.21 (13.90 to 14.51) |
| age | 0.08 (-0.06 to 0.22) |
| sMLH | -0.05 (-0.16 to 0.05) |
| aviary set-up (with females) | 0.13 (-0.17 to 0.43) |
| year (2015) | -0.62 (-0.92 to -0.32) |
| **Random effects** |  |
| male ID | 0.21 (0.16 to 0.27) |
| aviary | 0.04 (0.02 to 0.08) |
| sample ID | 0.16 (0.13 to 0.19) |
| residual variance | 0.86 (0.83 to 0.88) |

| **Table S1. continued** |  |
| --- | --- |
| **Sperm length (μm)** | |
| **Captive house sparrows** | estimate (lower CrI to upper CrI) |
| **c) midpiece** |  |
| (intercept) | 66.75 (66.09 to 67.39) |
| age | -0.03 (-0.39 to 0.34) |
| sMLH | 0.03 (-0.32 to 0.40) |
| aviary set-up (with females) | 0.67 (0.14 to 1.19) |
| year (2015) | 0.77 (0.19 to 1.34) |
| **Random effects** |  |
| male ID | 2.95 (2.34 to 3.63) |
| aviary | 0 (0 to 0) |
| sample ID | 1.24 (0.99 to 1.54) |
| residual variance | 2.36 (2.29 to 2.43) |
|  |  |
| **d) flagellum** |  |
| (intercept) | 85.61 (84.86 to 86.34) |
| age | 0.10 (-0.38 to 0.55) |
| sMLH | -0.13 (-0.57 to 0.30) |
| aviary set-up (with females) | 0.65 (0.04 to 1.27) |
| year (2015) | 0.03 (-0.61 to 0.62) |
| **Random effects** |  |
| male ID | 7.29 (5.76 to 8.97) |
| aviary | 0.05 (0.02 to 0.10) |
| sample ID | 0.47 (0.36 to 0.59) |
| residual variance | 2.87 (2.78 to 2.96) |

We accounted for sMLH, aviary set-up (levels: with, without females) and year of sperm collection (levels: 2014, 2015). Male age, as well as sMLH were centred and scaled. We present posterior means and CrI.

**Table S2.** Results from a generalized linear mixed model on the proportion of morphologically abnormal sperm in relation to male age in captive house sparrows (51 samples of 38 males) screened with one microscope only.

| **Proportion of morphologically abnormal sperm (logit-link scale)** | |
| --- | --- |
| **Captive house sparrows** | estimate (lower CrI to upper CrI) |
| (intercept) | -2.12 (-2.57 to -1.65) |
| age | 0.16 (-0.13 to 0.44) |
| sMLH | -0.13 (-0.44 to 0.17) |
| aviary set-up (with females) | -0.14 (-0.99 to 0.74) |
| method (faeces) | -0.27 (-0.77 to 0.25) |
| **Random effects** |  |
| male ID | 0.39 (0.23 to 0.59) |
| aviary | 0 (0 to 0) |
| observation-level random factor | 0.47 (0.31 to 0.66) |
|  |  |

We accounted for sMLH, aviary set-up (levels: with, without females), and sperm collection method (levels: abdominal massage, faeces). Male age, as well as sMLH were centred and scaled. We present posterior means and CrI.

**Table S3.** Results from a linear mixed model on cloacal protuberance volume (mm^3^) in relation to male age in wild house sparrows (*N* = 46 males) excluding repeated measurements.

| **cloacal protuberance volume (mm^3^)** | |
| --- | --- |
| **Wild house sparrows** | estimate (lower CrI to upper CrI) |
| (intercept) | 3.29 (3.06 to 3.54) |
| age | 0.10 (-0.09 to 0.29) |
| day of year | -0.43 (-0.67 to -0.19) |
| tarsus | -0.04 (-0.23 to 0.13) |
| year (2016) | -0.31 (-0.80 to 0.19) |
|  |  |

Using male ID as a random effect resulted in zero estimated variance signalling too few repeated measurements from males. To ensure that our main model was robust, we re-ran it using one randomly selected observation (function sample in R version 3.5.3 (‘R Development Core Team’, 2013) per male only. Cloacal protuberance volume was log-transformed. Male age, day of the year and tarsus length were centred and scaled continuous input variables. We present posterior means and CrI.

**Table S4.** Results from a generalized linear mixed model on the number of sperm at the perivitelline layer of 40 eggs from 9 aviaries in relation to male and female age (levels: old, young) in captive house sparrows. The identity of males and females was unknown meaning that we cannot recognise non-independence of data in this analysis, e.g. multiple measurements from the same bird.

| **Proportion of morphologically abnormal sperm (logit-link scale)** | |
| --- | --- |
| **Captive house sparrows** | estimate (lower CrI to upper CrI) |
| (intercept) | 4.53 (3.90 to 5.16) |
| male age (young) | -1.19 (-2.06 to -0.29) |
| female age | 0 (-0.83 to 0.76) |
| **Random effects** |  |
| aviary | 0 (0 to 0) |
| observation-level random factor | 1.83 (1.39 to 2.47) |
|  |  |

Unincubated eggs were collected from captive females that were either held in aviaries with only old males (seven and eight years old), or young males (one and three years old). We present posterior means and CrI. We added an observation-level random effect to account for overdispersion.
